# A manually annotated corpus in French for the study of urbanization and the natural risk prevention

**DOI:** 10.1038/s41597-023-02705-y

**Published:** 2023-11-22

**Authors:** Maksim Koptelov, Margaux Holveck, Bruno Cremilleux, Justine Reynaud, Mathieu Roche, Maguelonne Teisseire

**Affiliations:** 1 UNICAEN, ENSICAEN, CNRS – UMR GREYC, 14000 Caen, France; 2 INRAE, F-34398 Montpellier, France; 3 UMR TETIS, Univ. Montpellier, AgroParisTech, CIRAD, CNRS, INRAE, Montpellier, 34090 France; 4 ICube, Université de Strasbourg, 67412 Illkirch, France; 5 French Agricultural Research for Development (CIRAD), Montpellier, France

**Keywords:** Developing world, Environmental impact

## Abstract

Land artificialization is a serious problem of civilization. Urban planning and natural risk management are aimed to improve it. In France, these practices operate the Local Land Plans (PLU – Plan Local d’Urbanisme) and the Natural risk prevention plans (PPRn – Plan de Prévention des Risques naturels) containing land use rules. To facilitate automatic extraction of the rules, we manually annotated a number of those documents concerning Montpellier, a rapidly evolving agglomeration exposed to natural risks. We defined a format for labeled examples in which each entry includes title and subtitle. In addition, we proposed a hierarchical representation of class labels to generalize the use of our corpus. Our corpus, consisting of 1934 textual segments, each of which labeled by one of the 4 classes (Verifiable, Non-verifiable, Informative and Not pertinent) is the first corpus in the French language in the fields of urban planning and natural risk management. Along with presenting the corpus, we tested a state-of-the-art approach for text classification to demonstrate its usability for automatic rule extraction.

## Background & Summary

Land artificialization is defined as the permanent change in all or part of the ecological functions of the soil, in particular its biological, water and climatic functions, as well as its agronomic potential, as a result of its occupation or use^1^. The definition of artificialized land involves all non-agricultural, non-wooded and non-natural land and concerns the areas which form the basis of human life^2^. However, land artificialization meets the needs of human society by satisfying housing demands, industrial and service infrastructures. These human activities tend to be concentrated in towns, which continue to spread^3,4^.

Land artificialization, a direct consequence of urban expansion, is one of the major causes of biodiversity erosion^5,6^ and climate change on our planet today^7^. As an example, any transformation of a natural area could lead to disappearance of plants^8^ or animals from the area. At the same time, artificial soil no longer absorbs CO_2_, and thus it also contributes to the increase in global warming^7^. The increase in artificialization occurs in the absence of densification of already artificialized land. In spite of the fact that artificial soil no longer absorbs CO_2_, the expansion of cities requires additional energy-intensive infrastructure (urban and service networks, transportation etc.), which only further increases the negative impact on the environment^9^. In addition, land artificialization increases the risks of natural disasters^10^. By definition, a sealed ground does not absorb rainwater, and in case of heavy rains the risk of flooding is amplified^11^. In order to reduce the impacts of artificialization there must be control over this process^12^.

Land is a non-renewable resource. Once land is sealed, it is lost permanently, and we cannot reverse the process. The key concern is how to control the expansion of artificial land use and mitigate its effects while meeting all our needs? General approach for achieving this is based on the following principles:

1. New areas must be artificialized more responsibly
2. Already urbanized sectors (vacant housing, industrial or commercial wasteland etc.) must be reused as much as possible
3. Existing areas must be redeveloped in a more compact and effective way

Urban planning and territory development play a crucial role in this process. These practices are reflected in planning documents, which establish planning rules applied to new development and major development constraints. These rules concern, for example, the height of buildings, their use, requirements for surrounding areas and public services. They also regulate the maximum permitted density in the area for new and existing constructions. As for the constraints, they provide information on public spaces, natural parks, infrastructure areas, historic sites and monuments.

To facilitate automatic verification of those rules on real territories, the rules must be in a form that can be processed by a computerized system. For that purpose, the rules must be extracted first from the existing planning documents. They can be verified then using, for example, spatial images. The numerous satellites observe our planet on a daily basis producing a lot of those images^13^, and thus there should be no problem in using this source of information. The main challenge of the problem is to develop an approach to automate processing of the information coming from the both. There is no typical solution for that up to now, and our project, Hérelles (https://anr.fr/Projet-ANR-20-CE23-0022) funded by the French national research agency ANR (Agence Nationale de la Recherche: https://anr.fr), aims to fulfill this shortcoming.

**Fig. 1:**
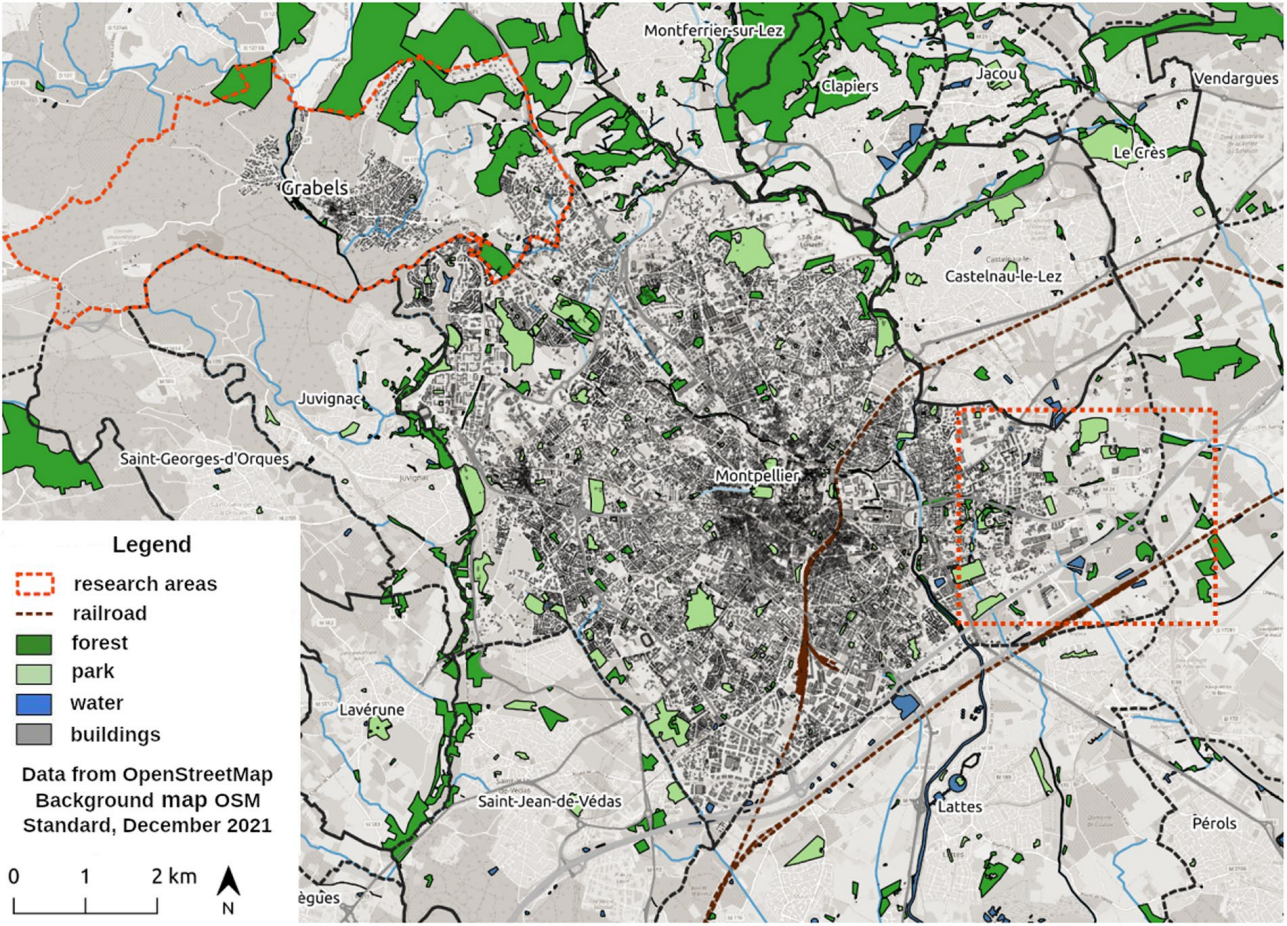
The geographical research areas in the 3M Metropolis in France: the area around the new railway station, Sud de France (South-East), and the Gimmel district of the Grabels municipality (North-West).

The application domains of Hérelles are the study of land artificialization and natural risk management. Geographically, the main case study of the project concerns Montpellier Méditerranée Metropolis (3M) in France, more precisely two sites in particular: (1) the area around the new railway station, Sud de France, at the south-east of the city of Montpellier; (2) the Gimmel district of the Grabels municipality, which is close enough to the borders of Montpellier (Fig. 1). These territories have been chosen for a number of reasons:

1. 3M is a fairly representative large metropolis in France with the necessary territorial development, given the constant growth of the population.
2. The area around the new railway station and the Gimmel district are selected for their recent evolution including major transport projects as well as for their environmental biodiversity and presence of natural risks.
3. Some members of our team working on the study have expert knowledge of the area and its evolution, which helps to analyze the results.

We believe that our project will help to save natural lands from artificialization by providing better management of an already artificialized territory. We try to achieve the latter by developing a solution for automatic verification of existing rules and constraints retrieved from urban planning documents.

**Fig. 2:**
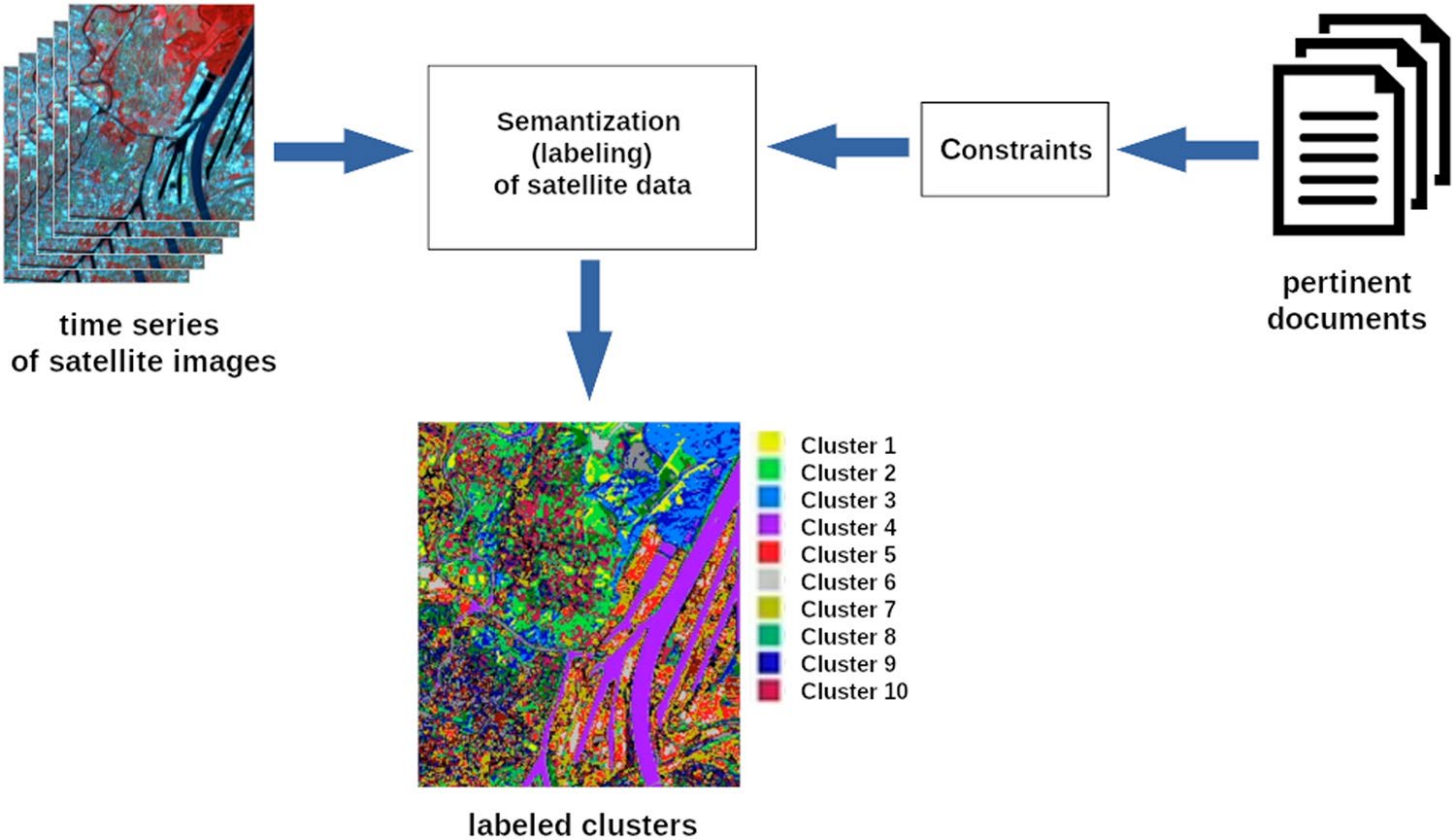
Schematic representation of the framework for collaborative clustering. Labeled clusters on the bottom image represent geographical objects detected in an unsupervised manner: water (Cluster 4), forestry and wooded areas (Cluster 3), port and industrial area (Cluster 5), urban housing (Cluster 6) and others (exact labels are assigned by the user of the system).

The final goal of our project is to develop a multimodal framework for *collaborative clustering*^14^. Collaborative clustering combines multiple clustering solutions (including different techniques, parameters and/or initializations) to group similar data points for a more reliable result. We benefit from using the collaborative setting to gain a more comprehensive and diverse perspective on the data. In the framework, the clusters are derived from the time series of satellite images. They are used then to verify the constraints contained in the regulatory documents (Fig. 2). To achieve that we associate textual elements of interest, which must respect research topics of the study and the spatio-temporal perimeter of the time series, with labeled (semanticized) clusters. In the following, we present the methodology implemented to identify and extract these textual elements of interest called *segments*. The resulting data set that we present in this article is aimed to facilitate the automatic extraction of segments from new planning documents.

The first step in our framework consists of extracting rules from text resources. In France, land artificialization and natural risk management practices are constrained by planning documents, in particular the Local Land Plans (PLU – *le Plan Local d’Urbanisme*) and the Natural risk prevention plans (PPRn – *le Plan de Pr*é*vention des Risques naturels*). These documents are written in French and they contain authorizations, obligations and prohibitions regarding land use and development. The PLU determines development plans and planning rules for the commune and its specific sites. The PLU divides a commune into zones by distinguishing between 4 types of land use: urbanized areas (Zone U), areas to be urbanized whose urbanization is planned (Zone AU), agricultural areas (Zone A) and natural areas (Zone N). The PPRn, in turn, regulates the land use and protects it from natural risks such as floods and fires.

**Fig. 3:**
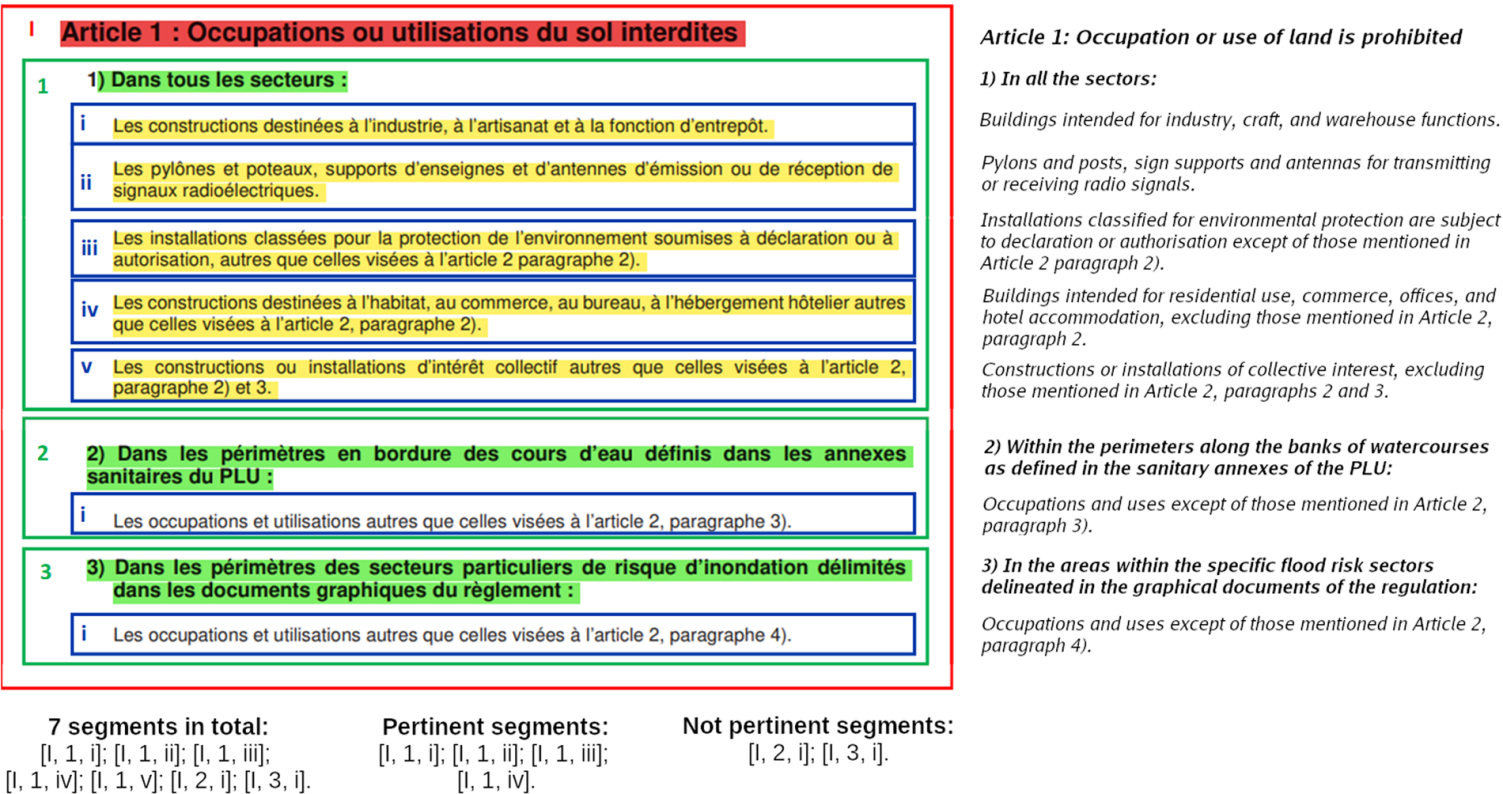
An extract from the PLU ZONE-5AU document (on the left), its translation to English (on the right) and the segments constructed from it (on the bottom). The title is highlighted in red, subtitles are in green and pertinent rules are in yellow.

In order to extract rules from the PLU and PPRn documents in an automatic manner a machine learning method can be employed. A common approach for automatic rule extraction from texts consists of exploiting a supervised learning setting^15,16^. The data must be segmented (i.e. split into segments) and labeled, a classifier is trained then using labeled examples. There are two main challenges in this modeling: how to represent the data and how to actually label it. A segmentation can be performed on the level of words^17^, sentences^18,19^ and fragments^20,21^ (parts of text with any number of sentences). Fragment representation better suits our needs, because the rules of our interest can be longer than one sentence. To use this method we define a *fragment* as a part of a document separated by one or more empty lines. In addition, we propose a special format to construct segments. Each *segment* in our representation consists of several fragments: a title, a subtitle and a part of the text with a potential rule (Fig. 3). The following example illustrates how a segment is constructed. The sentence “Les installations classées pour la protection de l’environnement soumises à déclaration ou à autorisation, autre que celles visées à l’article 2 paragraph 2)” (In English: “Installations classified for environmental protection are subject to declaration or authorisation except of those mentioned in Article 2 paragraph 2).”) taken out of context does not present any useful rule. By adding the subtitle “Dans tous les secteurs [de la zone 5 AU]” (In English: “In all the sectors [of the 5 AU zone]”), the spatial information where the rule must be applied is stated. Finally, the title “Occupations ou utilisations du sols interdites” (In English: “Occupation or use of land is prohibited”) indicates the type of the rule, prohibition in this case (Fig. 3). Our preliminary experiments without this type of formatting (i.e. where each fragment is used as a separate segment) demonstrated low performance of the model. Moreover, taking into account the structure of the documents used, a lot of information concerning the type of constraint and the spatio-temporal characteristics of the rules are located in the titles and subtitles of the selected documents. Segments not containing this information, therefore, are not very useful for labeling the clusters derived from time series of satellite images.

**Fig. 4:**
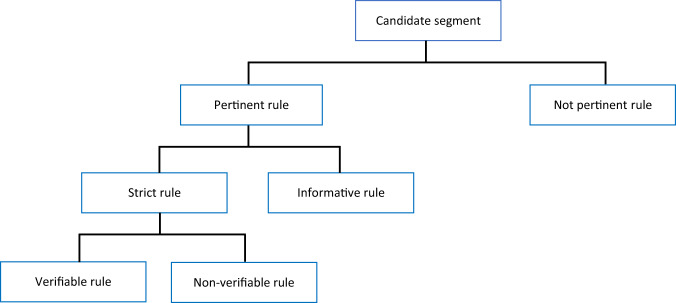
Hierarchical representation of segments with rules (pertinent) and without (not pertinent).

Taking into account that different types of rules have different importance and some of them are unlikely useful for general purposes, the labeling process is not an easy part either. To solve this problem we proposed a hierarchical representation of segments with multiple classes. In total we have defined 6 types of segments: pertinent, not pertinent, strict, informative, verifiable and not verifiable (Fig. 4). We call a segment *pertinent* if it contains information related to our research topics, respects the spatio-temporality principle and satisfies other criteria (Table 1). We determine the thematic adequacy by the presence of words from a *nomenclature* in the rule. A nomenclature is a collection of thematic concepts describing the research topics allowing to identify the different geographical objects observable in the satellite data. For instance, “chemin de fer” (railroad in English), “stationnement” (parking), “jardin public” (public garden) and “forêt” (forest) are examples of such concepts. Our manually curated *H*é*relles nomenclature* (10.57745/OXACT8), which is based on two existing nomenclatures, the FoDoMust project nomenclature^22^ and the Artisols project nomenclature^23^, contains 67 of those concepts. As for *spatio-temporality*, a pertinent segment must contain information about the research area and a time frame (or this information must be easily retrievable from the document description). A segment is called *not pertinent* if it does not contain this information. Not pertinent rules might be reminders of the law or definitions, elements which do not correspond to the scope of study, but also layout elements, bibliography, headers and footers, etc. (Table 1). For example, there are no nomenclature concepts in the sentence “Les volets doivent être réalisés en bois peint” (In English: “The window shutters must be made of painted wood”) and thus it is not pertinent. We call a segment *strict* if it describes an obligation, prohibition or authorization in a juridical manner and there is no ambiguity w.r.t. its application (i.e. the strict rule clearly states what must be done, what is prohibited or what is authorized). For example: “Dans l’ensemble de la zone les secteurs N-1, N-2, N-3, N-4, N-5 sont interdits : Les constructions destinées à l’habitation” (In English: “Throughout the territory in sectors N-1, N-2, N-3, N-4, N-5 are prohibited: Buildings intended for living”) is a strict rule, because it has a clear prohibition. We call a segment *informative* if it does not have a juridical value but provides additional details on the research area and the study topics which allow a better understanding of them. Informative segments often have the form of a definition or recommendation. For example: “La superficie habituellement affectée à chaque emplacement [de stationnement], accès directs inclus, est d’environ 25 *m*^2^” (In English: “Usually, the surface of allocated area to each [parking] location, direct accesses included, is approximately 25 *m*^2^”) is a recommendation. Finally, a segment is *verifiable* if it is possible to verify by satellite images and *not verifiable* otherwise. This distinction is important for the selection of constraints in the next steps of the project. For example, information related to the height of objects is impossible to verify by images, e.g. a height of a building or a construction (Table 1). These definitions were initiated by a specialist in the field (the second author of the article) and verified by the geographers involved in the project. The main idea behind such multi-level representation is that one could choose a level of their interest: by classifying segments on pertinent and not, by selecting strict, informative and not pertinent or by exploiting 4 non overlapping categories corresponding to the leaves of our hierarchy (verifiable, not verifiable, informative and not pertinent).

**Table 1:** Characteristics of pertinent rules.

Criteria	Details	Examples
Thematic fit	Consideration of the rules concerning land use, natural risk management and their regulations	Example of a thematic rule: *“Les constructions destin*é*es à l’habitation sont admises…”* (“Constructions intended for habitation are permitted…”) Example of a rule out of the scope of the study: *“Les volets doivent être r*é*alis*é*s en bois peint.”* (“The shutters must be made of painted wood.”)
Spatio-temporal adequacy	Only regulations respecting research sites for a chosen period are considered	Example of data respecting research sites: *“R*è*glement zone N à Montpellier”* (“Regulation zone N in Montpellier”) Example of data outside the research sites: *“R*è*glement zone UA du PLU de Montpellier”* (“UA zone regulation of the PLU of Montpellier”)
Rule observability	The application of the rules must be verifiable by satellite images	Example of an observable rule: *“Sont interdit: toute nouvelle construction, sauf l’extension des bâtiments existants.”* (“Prohibited: any new construction, except for the extension of existing buildings.”) Example of a non observable rule: *“Les arbres de hautes tiges existants et les masses v*é*g*é*tales significatives, doivent être maintenus…”* (“Existing tall trees and significant plant masses should be maintained…”)
Explicit data	The rules must be unambiguous. All the elements necessary for their use must be present (i.e. no reference to other documents). References to another paragraph or a chapter of the same document are considered explicit	Example of an explicit rule: “*Tous travaux, de quelque nature qu’ils soient, à l’exception de ceux vis*é*s au paragraphe ci-dessous…”* (“All works of any nature except of those specified in the paragraph below…”) Example of a non explicit rule: *“Sont admis: les changements de destination […] dans l’emprise existante des bâtiments rep*é*r*é*s sur les documents graphiques du r*è*glement au titre de l’article L. 15111 du code de l’urbanisme”* (“Allowed: changes of destination […] in the existing footprint of the buildings identified on the graphic documents of the regulations under article L. 15111 of the town planning code.”)
Text data	Consideration of textual rules only (e.g. diagrams are not considered due to the difficulty of their processing)	Example of textual data taken into account: *“Sont admis: les constructions à usage d’habitation…”* (“Are admitted: constructions for residential use…”) Example of a rule not taken into account: Rule concerning easements identified on the plan (which is present in a figure)

We refer to the corpus that we constructed as **LUPAN (Local Urban Plans And Natural risks)**. We manually labeled only verifiable, non-verifiable and informative rules. All the rules which are not labeled are considered to be not pertinent. Strict rules can be deduced by combining verifiable and non-verifiable rules together. In the same way pertinent rules can be derived, by combining strict and informative. We used 2 types of documents to construct our corpus: the PLU and the Natural flood risk prevention plans (PPRI – *le Plan de Pr*é*vention des Risques naturels d’Inondation*), which is a particular case of the PPRn. We only used documents concerning our research areas in the 3M Metropolis mentioned before (Fig. 1). In total we have managed to extract and label 1934 segments from 9 of those documents. Detailed statistics on each type of segment and each document are provided in Table 2.

**Table 2:** Number of segments corresponding to each class and each document.

Document	Number of segments						
	Pertinent					Not pertinent	Total
	Strict			Informative	Total		
	Verifiable	Non-verifiable	Total				
PLU ZONE-A	5	27	32	8	40	31	71
PLU ZONE-N	9	40	49	25	74	51	125
PLU ZONE-AU0	5	35	40	12	52	37	89
PLU ZONE-14AU	5	22	27	16	43	40	83
PLU ZONE-5AU	4	25	29	13	42	52	94
PLU ZONE-4AU1	6	66	72	21	93	73	166
PPRI Règlement Montpellier	12	77	89	16	105	20	125
PPRI Règlement Grabels	12	47	59	25	84	15	99
PLU Règlement Grabels	50	212	262	96	358	724	1082
TOTAL	108	551	659	232	891	1043	1934

To the best of our knowledge, LUPAN is the first labeled corpus in French in open access on the topics of urban planning and natural risk management. It should facilitate development of methods for automated extraction of rules from regulatory documents in the French language. Moreover, annotated text data are much rarer in French than in English (on the date of submission, there are 55 data sets in the French language in the HuggingFace library^24^, one of the biggest collections of publicly available data for NLP, comparing to 525 data sets in English, and only 12 of those sets are multi-label comparing to 152 analogous sets in English), and existing data in the former are mostly related to the biomedical field^25–27^ or to more traditional topics such as sentiment analysis^28,29^. We believe that the researchers working on French text mining, especially on multi-label classification, should find our corpus useful for testing their implementations. For this reason, in addition to the corpus, we present a preliminary evaluation of LUPAN with a state-of-the-art classification method in the Technical validation section. The results of the latter can be used for benchmarking by those researchers.

## Methods

**Fig. 5:**
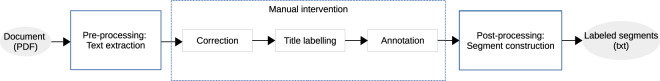
The workflow presenting different steps in construction of labeled segments.

In this section, we detail the steps we followed to construct the LUPAN corpus: text extraction, manual intervention and segment construction. The overview of this process is presented in Fig. 5.

### Text extraction

The PLU and PPRI documents originally come in PDF (Portable Document Format: https://www.adobe.com/acrobat/about-adobe-pdf.html). Our first step, therefore, was extraction of the text from the PDF files. It was implemented with the *fitz* module of the PyMuPDF library (https://github.com/pymupdf/PyMuPDF) in Python. Each document separated into pages was converted into a plain text file using that library. The code is available in our code repository, which is present in the Code Availability section below.

To construct our corpus we used the following sources of the PLU and PPRI documents: https://www.montpellier.fr/2300-reglement-du-plu-pieces-ecrites.htm (ZONE-A, N, AU0, 14AU, 5AU, 4AU1) as a source of PLU Montpellier, http://www.ville-grabels.fr/1637-environnnement.htm (Plan Local d’Urbanisme (PLU) approuvé de la ville de Grabels > IV – Règlement) as a source of PLU Grabel and https://www.herault.gouv.fr/Actions-de-l-Etat (Environnement, eau, chasse, risques naturels et technologiques > Risques naturels et technologiques > Les Plans de Prévention des Risques approuvés > Dossiers des PPR approuvés au format PDF > PPR Inondation > Règlement) as a source of PPRI Montpellier and PPRI Grabel.

### Manual intervention

In this step, we manually corrected files produced in the previous and performed their labeling and annotation. The protocol of manual intervention was developed in several phases using a small sample of fragments extracted from one document. All the documents have been checked to ensure that their format is the same: titles, subtitles and potential rules are separated by empty lines, the type of the rule and spatio-temporal information can be derived from titles and subtitles. After each phase we collected feedback from the members of our team and adjusted the protocol when it was necessary. As a result, some definitions have been reformulated and specified. A fragment annotation algorithm has been developed (see the Annotation section). Once the process was stabilized, the protocol was validated and applied to the rest of our corpus.

In the initial phase, we tried different orders of segment annotation and title labeling. The following analysis showed that annotated titles and subtitles are impractical and therefore there is a need to optimize the protocol. At the same time, we tried to annotate fragments in 2 steps: by labeling only pertinent ones first and relabeling pertinent with more classes after. We found that using a two-step annotation process did not provide a time-saving benefit, which led us to simplify the procedure to a one-step approach in which each corresponding fragment is directly annotated as belonging to one of the classes. In the following, we detail the final version of the protocol.

#### Correction

Our main intervention involved proper splitting of the text into fragments. For that we formally defined a *fragment* as a part of the text separated by two empty lines at most. In addition, we performed cleaning of the documents, for which we removed the tables of contents and figure descriptions wherever applicable.

#### Title labeling

Next, we manually labeled fragments corresponding to the titles and subtitles w.r.t. the original documents in the PDF format. We used the predefined sets of special characters for that, which we detail in Annotated document format below. This process was realized by a domain specialist and took from two to three hours for all documents in total.

#### Annotation

The annotation methodology has been developed as follows. An initial identification of the rules was carried out by the expert. The question of the modality of the rule then arose according to the associated spatial information. This led to a refinement of the annotations. For example, the sentence “les installations classées pour la protection de l’environnement” (In English: “installations classified for the protection of the environment”) may designate a prohibition or an authorization depending on the location of the rule and the relevant area. By adding the title (In English: “Article 2: Land occupations or uses subject to special conditions”) and subtitles (In English: “In sectors N-1, N-2, N-3, N-4, N-5”, “Are admitted”) of the article, the rule becomes explicit:


Article 2 : Occupations ou utilisations du sol soumises à des conditions particulièresDans les secteurs N-1, N-2, N-3, N-4, N-5 :Sont admises […] :• les installations classées pour la protection de l’environnement…


Finally, the methodology has evolved in order to classify the rules according to several classes (more than just splitting them on pertinent and not) and to avoid any ambiguities. As a result, we developed an algorithm for fragment annotation, which we present below. We used this algorithm in the last step of the manual intervention to annotate the rest of the fragments, which are not titles nor subtitles.

In order to verify our annotation methodology, a subset of segments was re-annotated by another member of our team. We used 296 segments (15% of all corpus) coming from 3 different documents: PPRI Règlement Grabels, PLU ZONE-A and PLU ZONE-N. We used only a subset of segments as considering the whole corpus would be too costly in human time. We have chosen PPRI Règlement Grabels and PLU ZONE-A as the shortest and most representative for each of the document type (PPRI and PLU), and we used PLU ZONE-N to introduce some diversity. After processing the results we found some inconsistencies in our definitions and the annotation algorithm. As a consequence, we updated the problematic definitions and our annotation algorithm. In the new version of the algorithm, we mark fragments as belonging to one of the following classes: Informative, Verifiable and Non-verifiable, which corresponds to the leaves of the hierarchy in Fig. 4. The final version of the algorithm can be summarized as follows:

1. If a rule candidate in combination with its title and subtitles satisfies the *thematic fit*, the *spatio-temporal adequacy* and the *explicit data* criteria defined in Table 1, we consider it to be *pertinent*. Otherwise, the rule is *not pertinent* and we **do not** annotate the fragment associated with it.
2. If a pertinent rule contains an obligation, prohibition or authorization, it is considered to be *strict*. Otherwise, the rule is not strict, and we thus **annotate** the corresponding fragment as **Informative**.
3. If a strict rule can be verified by satellite images the fragment associated with it is **annotated** as **Verifiable** and **Non-verifiable** otherwise.

As before, the use of special characters for annotation has been chosen accordingly (see Annotated document format).

Using an updated version of the fragment annotation algorithm we produced a new consolidated version of our corpus after which we repeated the re-annotation experiment as it was described above. The results of this new experiment are presented in Table 3. As can be seen from Table 3, the difference constitutes 5,0%. After analyzing differently annotated segments, we came to the conclusion that these segments are on the borderline between two classes. For example, the following rule can be interpreted as verifiable and not-verifiable at the same time: “swimming pool is at the level with the natural terrain is admitted”. It might happen because verifying with satellite images that a swimming pool is at the level with the natural terrain is complicated (part of the segment is non-verifiable), but at the same time the presence of the swimming pool is observable with satellite images (part of the segment is verifiable). Fortunately, the percentage of such ambiguous segments is not high. Moreover, their mislabeling is not critical since, as in the aforementioned situations, both interpretations are correct and they both fall to the pertinent category. To make a more reliable assessment we computed the kappa coefficient^30^ between these two annotations and we received 0.93 as the result. According to^31^, 0.81–0.99 corresponds to almost perfect agreement. We can conclude that our annotation methodology is reliable enough.

**Table 3:** Identically and differently annotated segments as a result of annotation methodology validation.

Document	Number of segments	Identically annotated by both annotators	Difference
PPRI Grabels	99	90	9
PLU ZONE-A	71	68	3
PLU ZONE-N	126	123	3
TOTAL	296	281	15

The rest of the annotation process was performed by the same specialist as in the previous step. However, this time it took seven working days to process all the documents in total taking into account the construction of the consolidation version of the corpus.

### Segment construction

At the end of our workflow, we performed automatic extraction of segments from the annotated documents. This step was also implemented in Python (see the Code Availability section).

In addition to automatic construction of segments using title and subtitle labels, our segment construction module is able to automatically detect and treat subsubtitles using simple set of rules:

- If a fragment is not labeled and it ends with ‘:’ then the fragment is a subsubtitle.
- If a fragment is not labeled and it starts with a digit or a letter followed by ‘)’ then the fragment is a subsubtitle.
- If a fragment ends with ‘:’ then the following fragment must start with a special identifier (a bullet point or a dash) otherwise the subsubtitle is not taken into account. For example:
  CONSTRUCTIONS ET OUVRAGES EXISTANTS :• Les aménagements ou adaptations visant à améliorer la sécurité des biens et des personnes.
- There is an exception if a subsubtitle starts with a digit or a letter followed by ‘)’ as in the example:
  c) ElectricitéLes branchements électricité, téléphone, vidéo-communication devront être établis en souterrain.
- Subsubtitle holds until there is a new title, subtitle or subsubtitle.
- Subsubtitle stops working when a type of an identifier changes or there is no an identifier as an example below:

Définition du prospect par rapport aux limites séparatives :Rappel : chaque prospect est calculé par rapport à la cote T.Ncorrespondante en limite séparative sur le fonds voisin.


The resulting segments are labeled by one of 4 classes: Verifiable, Non-verifiable, Informative and Not pertinent. The other two classes from Fig. 4 can be derived directly by combining Verifiable and Non-verifiable to get the class Strict, then by combining Strict and Informative to obtain the class Pertinent.

## Data Records

The presented data that we constructed are stored at Hérelles Dataverse (10.57745/XIVJ65)^32^. The corpus is presented by two files: *Corpus_Extracted_Segments_Consolidated_Version.zip* and *Corpus_Manual_Annotation_Consolidated_Version.zip*. The first file includes labeled segments and the second – an annotated corpus, which the segments were extracted from.

*Corpus_Extracted_Segments_Consolidated_Version.zip* contains 1934 labeled segments. Each file in the archive corresponds to the document using which the segments were constructed. The segments are stored as a plain text. In addition to the segments, we provide a manually annotated corpus itself. *Corpus_Manual_Annotation_Consolidated_Version.zip* contains an arxiv with 9 text files corresponding to 9 documents summarized in Table 2. These documents come from the PLU of Grabels, the PLU of Montpellier and the PPRI of Grabels. The name of the file consists of 2 parts: the type of the document (PLU or PPRn) and the name of the area (zone identifier or municipality name).

The formats of the files are described in detail in *Read_Me_Consolidated_Version.pdf*, and the links to the original documents used for constructing the corpus are presented in *Corpus_Expert_Links.tab*. The both of these files are included at Hérelles Dataverse.

### Annotated document format

The line with >>> p. indicates a page number starting from 0. Each fragment is separated by an empty line, and the first fragment is always a document name.

The fragments are annotated as follows: the character set *** represents a title, ** is a subtitle, ˆˆ is a verifiable rule, << is a non-verifiable rule and >> is an informative rule. The fragments which are not annotated are potential subsubtitles or not pertinent rules.

For better visual perception the parts of the text starting with a new title are separated by 2 empty lines.

### Segment file format

A new segment starts with >>> characters followed by a label name. There are 4 types of labels in total: False corresponding to not pertinent segments, Soft corresponding to informative, Verifiable and Non-verifiable to verifiable and non-verifiable respectively. A segment consists of fragments, each of which is separated by an empty line. There can be from 2 to 4 fragments in the segment. The first fragment is always the title and the last is always a rule. For example, a typical segment with 3 fragments has a title, a subtitle and a rule:


>>> FalseArticle 11 : Aspects extérieurDans l’ensemble de la zone :Toute expression architecturale est admise si elle répond à une qualité à la fois de conception (rythme, percements, proportions ou alternance pleins-vides) et à une cohérence architecturale de l’ensemble.


Presence of a subtitle is not mandatory, which results in the 2 fragment segment. For example:


>>> SoftZONE BLEUE “BU”Zone bleue “BU” : correspond aux zones inondables densément urbanisées exposées à des risques moindres (champs d’expansion des crues où les hauteurs d’eau pour la crue de référence sont inférieures à 0,50 m).


Finally, there might be a subsubtitle in the segment. In that case the segments will contain 4 fragments as in the example:


>>> Non-verifiableArticle 13 : Espaces libres et plantations3) Dans le secteur N-3 et N-6 :Règles :– Les aires de stationnement doivent être plantées à raison d’un arbre de haute tige pour 3 places de parking minimum, à l’exception des stationnements longitudinaux situés le long des voies privées qui feront l’objet d’aménagements spécifiques.


To improve visual perception, segments are separated by 2 empty lines.

## Technical Validation

In this section, we present evaluation results of LUPAN produced with a state-of-the-art classification method. First, we briefly discuss related work for segment classification. We continue by detailing our experimental setup. Finally, we present and discuss the results.

### Related work

Traditional approaches for segment classification are based on using specific measures originally coming from Information Retrieval (IR). As an example, a TF (term frequency) measure or its inverse version^33^ can be used for computing text features, the result of which can be used later in a machine learning model. In this type of approach, a classifier is trained on a labeled corpus, then predictions are performed on new unlabeled documents. A more advanced approach consists of using a Deep learning model, such as an LSTM (Long short-term memory) autoencoder^34^, for automatic deriving of features. The main disadvantage of this approach is in the amount of data which is required to properly train the model. Transfer learning^24^ is the most recent method which solves this problem. A transfer learning model allows *pre-training* a classifier on a larger corpus then a smaller corpus is used for *fine-tuning* the model. The most advanced approach in this category is the model of type BERT (Bidirectional Encoder Representations from Transformers)^35^, which is trained on a large corpus of webpages in English. For other languages there exist special extensions and CamemBERT^36^ is among them for the French language. The principal difference with BERT is that CamemBERT was trained on a large corpus in French, also parameters of the model were optimized accordingly. Another alternative for the French language is FlauBERT^37^, which was trained on a very large and heterogeneous French corpus. As a consequence, FlauBERT better suits for some specific tasks while CamemBERT remains to be more general. The most recent studies show that CamemBERT outperforms FlauBERT^38,39^. We thus select CamemBERT for evaluating LUPAN as a more general and best performing approach for segment classification.

### Prediction model

**Fig. 6:**
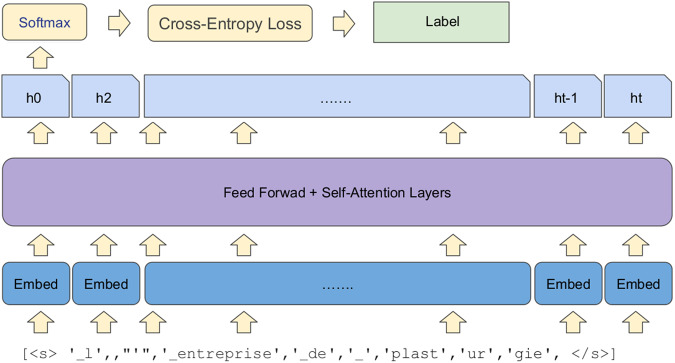
Schematic representation of an architecture of a BERT-type model^46^.

A model of type BERT is a stack of Transformer encoder layers^40^, consisting of several “heads” with self-attention (Fig. 6). For each input token in the sequence, each head computes the key, value, and query vectors used to create the weighted representation. The outputs of all heads of one layer are combined and pass through a fully connected layer. Each layer is wrapped with a skip connection, followed by layer normalization.

All the parameters of the model are trained to minimize a loss function for a given task. To get predictions of class labels a distribution over the labels is computed using the softmax function:

$$softmax(s)=\frac{{e}^{{s}_{i}}}{{\sum }_{k}{e}^{{s}_{k}}}{\rm{,}}i\in [| 1,K| ]{\rm{,}}s\in {{\mathbb{R}}}^{K}{\rm{,}}$$

where *s* – logit vector, *K* – number of class labels.

The learning process with a model of type BERT consists of the following phases: segmentation of an input text, initialization of the model parameters, pre-training and fine-tuning. In the first phase, sentencepiece tokenization^41^ is performed to segment the input text. To achieve that raw text must be segmented into modeling units or *tokens*. Special tokens are added to store the combined weights for classification predictions and to separate the input segments. Next, instead of randomly initializing all the parameters, the model is pre-trained on the masked word prediction task^35^. In this task, the model tries to predict the original vocabulary of the masked content based only on its context. This way of initializing the parameters prevents the model from overfitting, and also provides better generalization. The last phase, fine-tuning, consists in adding a text classifier on top of the final encoder layer. We use this phase to train CamemBERT further on our corpus for predicting proper labels of the input text.

### Experimental setting

#### Evaluation protocol

We evaluated LUPAN on a multi-label classification task. We classified each segment by belonging to one of the 4 classes: Verifiable, Non-verifiable, Informative and Not pertinent, which correspond to the leaves of the hierarchy of classes in Fig. 4. We first report results corresponding to the Pertinent vs Not pertinent classes and to Strict vs Informative w.r.t. our hierarchy in Fig. 4. They should provide a global vision of the performance of our corpus. We then report results corresponding to the leaves of the hierarchy to get a more detailed performance. As for the classification model, we employed CamemBERT as the most advanced method for text classification.

To perform evaluation of our experiments, we used stratified cross-validation implemented as follows. The data were split into 2 parts: 80% of segments were used for learning, and 20% were used for validation. The split was performed in such a way that the proportion of segments in each of the classes remains the same (Table 4).

**Table 4:** Number of segments per class in the corpus and in the training and test sets used for evaluation.

Class	Number of segments			Percentage
	Corpus	Training set	Test set	
Pertinent	891	713	178	46%
Strict	659	527	132	34%
Verifiable	108	86	22	6%
Non-verifiable	551	441	110	28%
Informative	232	186	46	12%
Not pertinent	1043	834	209	54%
TOTAL	1934	1547	387	100%

#### Quality measures

To assess the quality of our prediction we used Precision, Recall and F_1_ score as widely used metrics in the NLP community. We computed these measures for each of 6 classes in our corpus.

We defined precision for class *i* as the ratio of examples correctly classified as positives (belonging to class *i*) over all examples classified as positives:

$$Pre{c}_{i}=\frac{T{P}_{i}}{T{P}_{i}+F{P}_{i}}{\rm{,}}$$

where *TP*_*i*_ – true positives for class *i* (examples correctly classified as belonging to class *i*), *FP*_*i*_ – false positives (examples incorrectly classified as belonging to class *i*). Precision for class *i* measures whether a model is specific enough to mainly classify segments of class *i* as actually belonging to that class.

We defined recall for class *i* as the ratio of examples correctly classified as belonging to class *i* over all examples of class *i* in the test data:

$${{Rec}}_{i}=\frac{{{TP}}_{i}}{{{TP}}_{i}+{{FN}}_{i}},$$

where *FN*_*i*_ – false negatives for class *i* (examples incorrectly classified as not belonging to class *i*). Recall for class *i* measures whether a model is general enough to classify a large proportion of class *i* as actually belonging to that class.

In addition to Precision and Recall, we also used F_1_ score defined as a harmonic mean of the both:

$${F}_{{1}_{i}}=2\times \frac{Pre{c}_{i}\cdot Re{c}_{i}}{Pre{c}_{i}+Re{c}_{i}}{\rm{.}}$$

F_1_ score symmetrically represents both those measures in one metric.

Due to known instability of fine-tuning of a model of type BERT^42^ we repeated each experiment 10 times and reported best and average results. We used *mean* to compute average and weighted accuracy to determine which result among 10 experiments is the best. Accuracy is a most common metric which can assess the prediction quality of all classes simultaneously and its weighted version gives a more accurate assessment for unbalanced data sets. We defined accuracy as the ratio of examples correctly classified for each class over total number of predictions:

$$Acc=\frac{{\sum }_{i=1}^{n}T{P}_{i}}{{\sum }_{i=1}^{n}T{P}_{i}+F{P}_{i}}{\rm{,}}$$

where *n* – total number of classes (4 in our case). To derive a weighted version of accuracy we assign a classification cost of 1 to examples of over represented class (Not pertinent in our case) and cost *pos_cost*_*i*_ to examples of underrepresented classes (Verifiable, Non-verifiable and Informative), derived by:

$$pos\_cos{t}_{i}=\frac{| D| }{2\times | {N}_{i}| }{\rm{,}}$$

where |*D*| – number of examples of all classes, |*N*_*i*_| – number of examples of class *i*. We then perform evaluation based on the costs defined: *TP*_*i*_ and *FP*_*i*_ receive score *pos_cost*_*i*_ for every positive example in classes Verifiable, Non-verifiable and Informative w.r.t. their real classes, and score 1 for positives in class Not pertinent. We benefited from using weighted accuracy twice: to determine the best performing epoch in each experiment and to select the best result among 10 runs.

#### Implementation details

We implemented CamemBERT in Python 3.8 using the *CamembertForSequenceClassification* model from the HuggingFace library^24^. We used *AdamW*^43^ as the most common optimizer for this task with the parameters recommended in^35^: learning rate 2 × 10^−5^ and *ε* = 10 × 10^−8^. We also fixed the number of epochs to 10 and the batch size to 16. Finally, we used the scikit-learn library^44^ to implement Precision, Recall, F_1_ score and weighted version of accuracy.

### Experimental results

The results corresponding to general classes are presented in Table 5. The model provides good global performance which is demonstrated by Recall more than 90% for the Pertinent and Strict classes (92% and 95% respectively) and F_1_ score close to 90% for both of the classes (87% and 88% respectively).

**Table 5:** The global evaluation results with the state-of-the-art approach (CamemBERT) corresponding to the experiment with maximum weighted accuracy value.

Classification	Precision	Recall	F_1_ score
Pertinent vs Not pertinent	0.82	0.92	0.87
Strict vs Informative	0.82	0.95	0.88

The detailed performance w.r.t. the leaves of the hierarchy is presented in Table 6. As can be seen from the results, the Not pertinent class is the easiest one to predict, which is not surprising taking into account that it takes the biggest part of the corpus. Non-verifiable class is the next one in this comparison, which is not surprising taking into account that it is the second biggest class in the corpus. The last two classes, Informative and Verifiable, are positioned the last. They share quite similar performance around 85% of F_1_ score, which is rather good, but still not ideal. The task is challenging in particular because these classes are fairly precise specializations of the corresponding rules. Nevertheless, it gives room for improvement in the future experiments.

**Table 6:** The detailed evaluation results with the state-of-the-art approach (CamemBERT) averaged among 10 runs and best results corresponding to the experiment with maximum weighted accuracy value.

Class	Precision		Recall		F_1_ score		Accuracy (best)	Accuracy* (max)
	avg	best	avg	best	avg	best		
Verifiable	0.82	0.83	0.85	0.86	0.84	0.84	0.90	0.89
Non-verifiable	0.84	0.82	0.89	0.96	0.86	0.88		
Informative	0.83	0.85	0.83	0.85	0.83	0.85		
Not pertinent	0.95	0.98	0.91	0.89	0.93	0.93		

One phenomenon which is interesting, the average values of Precision for Non-verifiable class and Recall for Not pertinent class are higher than their best values. It can be explained by the fact that we use weighted accuracy to find an optimal experiment setting, and some individual measures in that experiment might not have maximum values. On the other hand, it would be interesting to test other metrics designed for assessing the quality of multi-label prediction. Among them are balanced accuracy, macro F_1_ score and their weighted versions^45^.

To validate our model using unseen data, we prepared an extract from the PLU of Strasbourg, another rapidly developing city in France, which is not included in the main corpus. Preliminary results on that data (i.e. PLU de l’Eurométropole de Strasbourg, CHAPITRE XIV-XVIII, XXIV) demonstrated good performance in general (overall accuracy 81%), but revealed that the Verifiable class has to be investigated in more detail. The lack of examples of this type introduces additional challenges to the learning process. Therefore, we would suggest trying to balance the corpus by artificially generating more examples for underrepresented classes and to perform a cascade classification with separate classifiers by Pertinent and Not pertinent, Strict and Informative, finally Verifiable and Non-verifiable classes. We intend to explore it in future work.

## Data Availability

The project framework that has been implemented for evaluating our corpus is stored at the corpus repository^32^ in the *LUPAN_code.zip* file: 10.57745/XIVJ65. It includes all the scripts used for constructing the corpus and the code of the preliminary experiments presented in Technical validation. The code has the following structure: 1. Corpus construction: • *pdf2text.py* – text extraction from the documents in the PDF format • *segment_construction.py* – segment construction from the annotated documents in the txt format 2. Preliminary experiments: • *data_loader.py* - load segments from the txt format, split the data into 80%/20% for learning/test • *segment_classification.py* – 4-label classification by the CamemBERT model To reconstruct the experiments: (a) Download and place the PDF documents to the *Corpus_PDF* folder. The links to the original documents are provided in the Text extraction section above. (b) Extract text from the documents using our *pdf2text.py* module. The resulting files will be saved to the *Corpus_txt* directory. (c) Manually pre-process the documents as it is decribed in Methods (annotation details) and Data records (data formats). To skip this part download *Corpus_Manual_Annotation_Consolidated_Version.zip* from the corpus repository, extract it to the same directory with the code. (d) Construct label segments from manually annotated documents using *segment_construction.py* module. To skip this part download *Corpus_Extracted_Segments_Consolidated_Version.zip* from the corpus repository, extract it to the same directory with the code. (e) Prepare label segments for learning by using *data_loader.py*. (f) Use *segment_classification.py* for learning and validation. For detailed examples please refer to the README file provided with the code.
